# The Pacific Ocean Virome (POV): A Marine Viral Metagenomic Dataset and Associated Protein Clusters for Quantitative Viral Ecology

**DOI:** 10.1371/journal.pone.0057355

**Published:** 2013-02-28

**Authors:** Bonnie L. Hurwitz, Matthew B. Sullivan

**Affiliations:** Ecology and Evolutionary Biology, University of Arizona, Tucson, Arizona, United States of America; Universidade Federal do Rio de Janeiro, Brazil

## Abstract

Bacteria and their viruses (phage) are fundamental drivers of many ecosystem processes including global biogeochemistry and horizontal gene transfer. While databases and resources for studying function in uncultured bacterial communities are relatively advanced, many fewer exist for their viral counterparts. The issue is largely technical in that the majority (often 90%) of viral sequences are functionally ‘unknown’ making viruses a virtually untapped resource of functional and physiological information. Here, we provide a community resource that organizes this unknown sequence space into 27 K high confidence protein clusters using 32 viral metagenomes from four biogeographic regions in the Pacific Ocean that vary by season, depth, and proximity to land, and include some of the first deep pelagic ocean viral metagenomes. These protein clusters more than double currently available viral protein clusters, including those from environmental datasets. Further, a protein cluster guided analysis of functional diversity revealed that richness decreased (*i*) from deep to surface waters, (*ii*) from winter to summer, (*iii*) and with distance from shore in surface waters only. These data provide a framework from which to draw on for future metadata-enabled functional inquiries of the vast viral unknown.

## Introduction

Bacteria are fundamental to life on Earth and drive energy and nutrient cycles in natural systems [1]. In the surface oceans, viruses, thought to be predominantly phages, and are present at approximately 10^7^ particles per milliliter of seawater often outnumbering their bacterial hosts fifteen to one [2]–[4]. These abundant phages play an integral role in the lifecycle, development and evolution of their diverse hosts (reviewed in [5]). This is particularly well documented in ocean cyanophages where natural abundances are high [6]–[9], and the eco-evolutionary virus-host dynamics involve manipulation of critical aspects of ocean life (e.g., photosynthesis, phosphate, nitrogen; reviewed in [10]). Despite this importance, our understanding of indigenous viral and microbial communities is compromised by both conceptual and technological challenges. Perhaps chief among them is that <1% of microbes in an environment can be routinely grown in the laboratory [11], [12].

To study this ‘unseen majority’ [13], microbial ecologists developed culture-independent techniques, first in the form of gene markers (e.g., small subunit ribosomal DNA, [14]) to quantify biodiversity and subsequently through metagenomics to study metabolic function and gene ecology [12], [15]–[17]. To this end, resources such as the Global Ocean Sampling (GOS) dataset [18] have been developed, and subsequently organized using sequence-based protein clusters (PCs) [19] to (*i*) group related proteins based on sequence similarity, (*ii*) map metadata associated with the samples to the PCs, and (*iii*) aid future inquires in genetic novelty by mapping to pre-defined PCs. The GOS dataset represents 7.7 million reads from 41 surface ocean microbial (0.1–0.8 µm size fraction) samples ranging 8,000 km along coastal Atlantic Ocean eastern United States waters to the Sargasso Sea, Gulf of Mexico, Caribbean Sea and a few sites in the South Pacific Ocean. This broad sampling has enabled GOS to greatly advance microbial ecology (849 citations as of 6 Sept 2012; Google Scholar) both through data mining studies and use of GOS to contextualize environmental research findings.

While metagenomics was first applied to uncultured viral communities [20], two years in advance of microbes [15]–[17], there remains no such equivalent GOS-scale datasets (Table 1). Such a resource is missing because of limiting DNA yields from environmental viruses and the fact that viral metagenomic analytics are challenging. On the latter, viral metagenomics is more difficult than microbial metagenomics as (*i*) viral genome representation in public databases is more sparse (as of November 2012 there are 115 marine phage genomes in Genbank, Table S1), (*ii*) analytical tools paralleling those for microbes (e.g., MG-RAST [21] and IMG/M [22]) have not been available (but see VIROME [23] and MetaVir [24]) and (*iii*) the ‘unknown’ problem is even greater in viral metagenomes as often 90% of sequences lack similarity to anything in pre-existing protein databases (see Table 1 versus 65% for microbes [17]). Worse, the viral metagenomic datasets themselves often suffer either from being too small (e.g., LASL-based Sanger-sequenced metagenomes) or contain technical artifacts that are now known to render the metagenomes non-quantitative. To emphasize the latter, we highlight a commonly used marine viral dataset (363 citations as of 6 Sept 2012; Google Scholar) that includes 1.77 million metagenome sequences from 4 major ocean regions [25]. While this dataset was generated using the best available technologies and is the gold-standard for contextualizing viral ecological findings, it is now known to suffer from two major technical issues: (*i*) pooled samples (4 metagenomes from 184 viral assemblages collected over a decade from 68 sites) inhibit tracking of variability over space and time, and (*ii*) MDA-amplified material is now known to lead to non-quantitative metagenomes [26]. This latter point is particularly problematic for viruses as MDA biases are both stochastic [26] and systematic [27], [28] with the latter related to nucleic acid type and structure (i.e., a feature which varies across viral types). For these reasons, there are currently *no* marine viral metagenomic datasets appropriate for quantitative viral ecology.

**Table 1:** Summary of published marine, non-coral viral metagenomic datasets.

site	Scripps Pier and Mission Bay,San Diego CA	Mission Bay, San Diego, CA	Jericho Pier andStrait of Georgia,British Columbia	BBC, GOM, ARC, SAR	Chesapeake Bay	Line Islands, Skan Bay	Tampa Bay, induced virome	Eutrophic MontereyBay, CA 200 m depth	East Pacific Risediffuse-flow viralcommunity	POV
**# metagenomes**	1	1	2	4	1	13	1	1	1	32
**Total # reads**	1 934	1 156	277	1 768 297	5 641	2 258 386	294 068	881	176	6 020 088
**avg. len. (bp)**	>600	>600	>600	102	695	<105	102	>600	488	310
**sequencing platform**	Sanger	Sanger	Sanger	454 GS20	Sanger	454 GS20	454 GS20	Sanger	Sanger	454 Titanium
**viral fraction (µm)**	<0.16	0.2	0.22	0.2	0.2	0.2	0.2	0.2	N/A	0.2
**concentration**	TFF	TFF	TFF+ultracent.	TFF	TFF	TFF	TFF	TFF	TFF+ultracent.	FeCl_3_-ppt
**purification**	CsCl	CsCl	RNAse	CsCl	N/A	CsCl	CsCl	CsCl	N/A	CsCl+DNase
**DNA amplification**	LASLs	LASLs	cDNA-based LASLs	MDA	LASLs	MDA	MDA	N/A	LASLs	LA
**% unknown**	65	75	63–81	91	70	90	93	74	75	89
**reference**	[20]	[73]	[74]	[25]	[41]	[42]	[75]	[30]	[43]	This publication

Abbreviations are as follows: TFF – Tangential Flow Filtration, ultracent. – ultracentrifugation, FeCl_3_-ppt – iron-chloride precipitation, CsCl – cesium chloride density gradient, LASLs – linker amplified shotgun libraries, MDA – multiple displacement amplification, LA – linker amplification.

Here we introduce a large-scale, quantitative Pacific Ocean Virome (POV) dataset and ∼456 K associated PCs that organize the ‘known’ and ‘unknown’ sequence space for future comparative viral metagenomic study. The 6 million read dataset is derived from 32 temporally- and spatially-resolved viral assemblages, and represents the largest viral metagenomic sampling of the Pacific Ocean to date, including the first large-scale viral metagenomes from the deep pelagic ocean (but see [29], [30] and Table 1).

The POV dataset represents a systematically collected, processed, and documented quantitative marine viral metagenomic resource [31] as follows. All thirty-two POV communities were concentrated using a new method that captures nearly all particles [32], purified using DNase digestion and CsCl buoyant density gradients to minimize contamination by non-viral DNA [33], and DNA extracted and linker-amplified to minimize quantitative and cloning biases in the resulting metagenomes [34]. DNA was then sequenced by Roche 454 Titanium technology. The metagenomes and the associated PCs provide a much-needed community resource to test hypotheses about environmental viruses, as GOS has done for microbial ecology. For these reasons, POV will likely become a foundational dataset for future comparative studies of virus genes and communities at the global ocean scale such as those derived from the recent *Tara Oceans* [35] and *Malaspina* [36] expeditions.

## Results and Discussion

### The Pacific Ocean Samples

**Figure 1:**
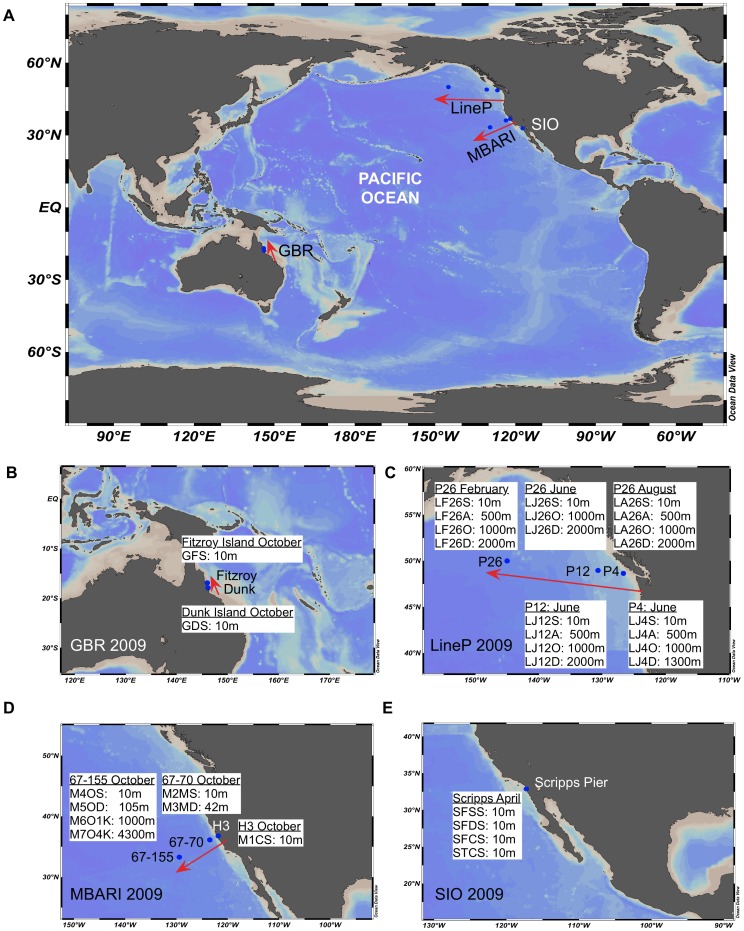
Sampling site map for the POV dataset. Thirty-two viral metagenomes represent discretely sampled and processed datasets that vary over time and space in the pelagic Pacific Ocean. (A) Overview of sampling sites, (B) GBR – Great Barrier Reef, Australia, near Dunk and Fitzroy Islands. (C) LineP- oceanographic transect off Vancouver Island, British Columbia (D) MBARI- Line67 oceanographic transect off of Monterey Bay, California (E) SIO- Scripps Pier, San Diego, CA. Images were created using Ocean Data View.

The 32 POV source waters varied by depth, proximity to land, and season and were derived from four regions in the Pacific Ocean (Figure 1): Scripps Pier in San Diego, California (SIO), Monterey Bay, California (MBARI), near Vancouver Island in British Columbia (LineP), and the Great Barrier Reef in Australia (GBR). Samples, metadata, and metagenomic descriptive statistics are summarized in Table 2.

**Table 2:** Description of POV samples and associated metadata.

sample	# reads	Mbps	Avg. read length	Stdev read length	photic zone	region	depth (m)	site location	month	season	Oceanic features	
L.Win.O.1000 m	147 537	40.5	274	80	aphotic	LineP	1000	open ocean	Feb.	winter	OMZ	
L.Win.O.2000 m	125 896	33.4	265	86	aphotic	LineP	2000	open ocean	Feb.	winter		
L.Spr.C.500 m	136 876	44.5	325	83	aphotic	LineP	500	coastal	Jun.	summer		
L.Spr.C.1000 m	97 126	31.5	324	124	aphotic	LineP	1000	coastal	Jun.	summer	OMZ	
L.Spr.C.1300 m	98 478	24.2	245	95	aphotic	LineP	1300	coastal	Jun.	summer		
L.Spr.I.500 m	58 108	20.5	353	101	aphotic	LineP	500	intermediate	Jun.	summer		
L.Spr.I.1000 m	122 565	45.9	374	88	aphotic	LineP	1000	intermediate	Jun.	summer	OMZ	
L.Spr.I.2000 m	49 914	13.2	264	97	aphotic	LineP	2000	intermediate	Jun.	summer		
L.Sum.O.500 m	42 118	13.6	322	122	aphotic	LineP	500	open ocean	Aug.	fall		
L.Sum.O.1000 m	70 596	19.9	282	131	aphotic	LineP	1000	open ocean	Aug.	fall	OMZ	
L.Sum.O.2000 m	68 516	18.6	271	122	aphotic	LineP	2000	open ocean	Aug.	fall		
L.Spr.O.1000 m	101 179	28.4	280	115	aphotic	LineP	1000	open ocean	Jun.	summer	OMZ	
L.Spr.O.2000 m	55 332	14.8	267	110	aphotic	LineP	2000	open ocean	Jun.	summer		
L.Win.O.500 m	167 616	48.0	286	84	aphotic	LineP	500	open ocean	Feb.	winter		
M.Fall.O.1000 m	225 833	66.3	293	105	aphotic	MBARI	1000	open ocean	Oct.	fall		
M.Fall.O.4300 m	144 588	40.5	279	95	aphotic	MBARI	4300	open ocean	Oct.	fall		
GD.Spr.C.8 m	116 855	29.0	247	72	photic	GBR	8	coastal	Oct.	spring		
GF.Spr.C.9 m	82 739	20.9	252	73	photic	GBR	9	coastal	Oct.	spring		
L.Sum.O.10 m	165 256	48.4	292	102	photic	LineP	10	open ocean	Aug.	fall		
L.Spr.C.10 m	107 244	25.7	240	86	photic	LineP	10	coastal	Jun.	summer		
L.Spr.I.10 m	92 415	22.6	244	92	photic	LineP	10	intermediate	Jun.	summer		
L.Spr.O.10 m	75 036	19.5	259	106	photic	LineP	10	open ocean	Jun.	summer		
L.Win.O.10 m	192 685	59.7	310	74	photic	LineP	10	open ocean	Feb.	winter		
M.Fall.C.10 m	303 519	105.2	346	118	photic	MBARI	10	coastal	Oct.	fall	Upwelling	
M.Fall.I.10 m	321 754	92.9	288	109	photic	MBARI	10	intermediate	Oct.	fall	Upwelling	
M.Fall.I.42 m	31 528	10.9	346	114	photic	MBARI	42	intermediate	Oct.	fall	Upwelling; DCM	
M.Fall.O.10 m	203 238	52.4	257	95	photic	MBARI	10	open ocean	Oct.	fall		
M.Fall.O.105 m	156 509	44	281	101	photic	MBARI	105	open ocean	Oct.	fall	DCM	
SFC.Spr.C.5 m	487 339	191.2	392	107	photic	SIO	5	coastal	Apr.	spring		
SFD.Spr.C.5 m	645 463	218.7	338	119	photic	SIO	5	coastal	Apr.	spring		
SFS.Spr.C.5 m	504 826	173.1	342	130	photic	SIO	5	coastal	Apr.	spring		
STC.Spr.C.5 m	821 404	246.3	299	103	photic	SIO	5	coastal	Apr.	spring		

Oceanic features include: OMZ = oxygen minimum zone, DCM = deep chlorophyll maximum, Upwelling = within a current system with upwelling.

The defining ecological features of each dataset are as follows. Four SIO metagenomes were derived from a single coastal, surface water sample from Scripps Pier (San Diego, CA, April 2009, spring), the site of the first viral metagenome [20], that were differentially concentrated and purified (4 treatments, >2.5 M sequences; [33]). Seven MBARI metagenomes (∼1.4 M sequences) represent viruses concentrated from various depths along a long-standing oceanographic transect, Line67 [33], [37], [38], which spans coastal, upwelling and open ocean waters off Monterey Bay, California collected in fall, October 2009. Nineteen LineP metagenomes (∼2.0 M sequences) represent viruses concentrated from various depths along another long-standing oceanographic transect, LineP [39], which spans coastal-to-open-ocean waters, including the second largest ocean oxygen minimum zone [40], off British Columbia collected in February (winter), June (spring) and August (summer) 2009. Finally, two GBR metagenomes (∼0.2 M sequences) represent viral concentrates from the dry season near Dunk (Tully River impacted) and Fitzroy (less impacted) Islands at the Great Barrier Reef in Australia collected in October (spring) 2009. Together, these metagenomes represent a diversity of pelagic ocean features including oceanic region (SIO, GBR, MBARI, LineP), proximity to land (coastal to open ocean), season (spring, summer, fall, and winter), depth (10 m to 4300 m), primary productivity and oxygen concentration (variability in other physiochemical characteristics are not considered here).

### Taxonomic Composition of POV Metagenomes

**Figure 2:**
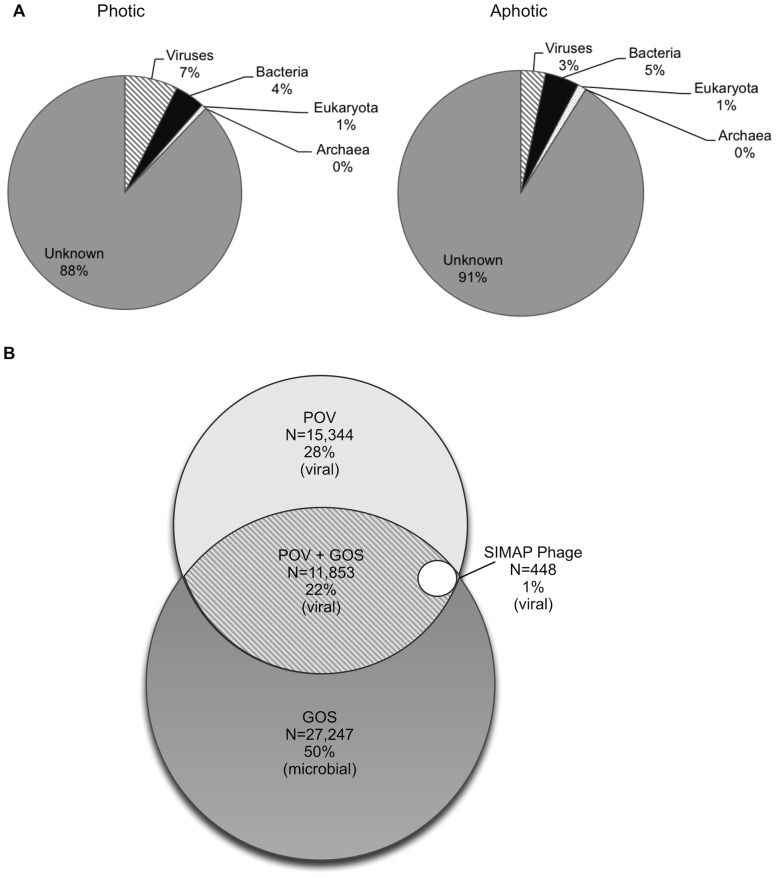
The POV dataset and its place in the viral protein universe. (A) Summary superkingdom taxonomy statistics for quantitative Pacific Ocean viral metagenomes from 16 photic and 16 aphotic zone samples. Reads were taxonomically assigned based on matches to proteins in SIMAP and curated as described in the methods. (B) Venn diagram representing medium- to large membership PCs documents the relative contributions of the POV, GOS microbial, and SIMAP datasets to the ‘viral protein universe’.

Long-standing questions in marine viral ecology are centered on understanding the extent to which viral assemblages change spatially, temporally and under different environmental conditions in the ocean [2]. Yet, given the paucity of known viruses in biological databases comparatively examining viral assemblages from diverse environments in the sea is stymied. As commonly observed in marine viral metagenomic studies [20], [25], [41]–[43], the majority (87% photic zone, 91% aphotic zone) of the reads could not be classified based on sequence similarity to known taxa (see Materials and Methods, Figure 2A). Moreover, we found a smaller fraction of reads that matched known viruses in the aphotic zone (3.3%) than the photic zone (8.3%) likely due to more sampling in the surface oceans (Figure 2A and Table 1).

To examine the taxonomic composition of the POV metagenomes in greater detail, we classified the reads from each sample based on their top match at the family level (Figure S1). Overall, we found that metagenomes from samples in the photic zone had a larger proportion of reads that matched *Myoviridae* (an average of 4.2%±1.9%) than in the aphotic zone (an average of 1.6%±0.8%). Several samples in the deep ocean however, were enriched for *Myoviridae* including L.Spr.I.2000 m (3.3%) and L.Spr.O.2000 m (3.0%) that closely matched their photic counterparts L.Spr.I.10 m (3.1%) and L.Spr.O.10 m (4.1%). Also notable were the large fraction of reads matching *Myoviridae* at the deep chlorophyll maximum (DCM) in the open ocean in Monterey Bay, (9.6% for M.Fall.O.105 m) which is more than four times the fraction seen in the surface ocean from the same time point and station (1.9% for M.Fall.O.10 m). We also found a large fraction of sequences that matched *Podoviridae* in the DCM sample in the open ocean in Monterey Bay (4.2% for M.Fall.O.105 m) and in the surface samples from the Great Barrier Reef (3.8% for GF.Spr.C.9 m and 5.0% for GD.Spr.C.8 m) as compared to the 0.8%±0.5% on average in other samples. Thus, *Podoviridae* may play an important role in reef ecosystems and the DCM not presently unknown.

Finally, we compared and contrasted known viruses at the genus and species level in the combined photic and aphotic samples (Figure S2A and B respectively). At the genus level, we found a higher fraction of T4- and T7-like viruses in the photic zone (6.9% total) than the aphotic zone (2.6% total). At the species level, we found a higher faction of *synechococcus* and *prochlorococcus* phages in the photic zone (4.6% total) than the aphotic zone (1.1% total).

### The Protein Cluster as a Means to Organize Unknown Sequence Space

While this great ‘unknown’ problem is exacerbated in viral metagenomes, it has also plagued microbial metagenomic studies to the extent that previous analyses of the GOS dataset organized this sequence space, including unknowns, using protein clustering (*sensu* Yooseph et al., 2007 and 2008 [19], [44]; details in Materials and Methods).

Here, as per Yooseph’s approach, we individually assembled each POV metagenome and identified open reading frames (ORFs) on both the contigs and individual reads, yielding ∼4.1 M non-redundant ORFs. These POV ORFs were clustered with ORFs from GOS core clusters (3,625,128 ORFs, [19] of both microbial and viral origin, as well as genes from SIMAP phage genomes (33,857 ORFs, [45] – in total ∼7.8 M ORFs. Given that database representation of viral sequences is sparse at best (e.g., GOS represents mostly microbial-fraction not viral core clusters) and the POV samples represent predominantly unexplored ocean regions, it is not surprising that most (78%) POV ORFs fail to cluster with known PCs (Table 3). Self-clustering the unmapped POV ORFs further organized this unknown sequence space (i.e., another 55% of POV ORFs were clustered), such that only 23% of POV ORFs remained as singletons. These singletons could either represent artifact or more likely are members of the “rare biosphere” [46] under-sampled in this data set due to their rarity.

**Table 3:** POV ORF recruitment.

photic zone	# ORFs	clustered with GOS/SIMAP phage	self-clustered	singletons	total clustered
photic	2 783 784	31%±4%	47%±9%	22%±7%	78%±7%
aphotic	1 323 811	13%±6%	62%±12%	25%±10%	75%±10%
all	4 107 595	22%±10%	55%±13%	23%±9%	77%±9%

The fraction of POV ORFs that non-redundantly recruited to existing (GOS/known phages) PCs versus those that recruited to PCs derived from the POV dataset (self clustered). POV ORFs are non-redundant by metagenome. The standard deviation values refer to differences in the fraction of ORFS clustered in the POV samples.

In total, we identified 456,420 PCs that contained two or more non-redundant members (12,226+1,557+442,637 PCs derived from GOS+POV, Phage+POV and POV only, respectively). Of these, 27,646 PCs contained 20 or more members (counts with varying levels of cluster membership are summarized in Table 4). For comparison, GOS, the first large-scale marine *microbial-fraction* metagenomic sequencing effort predicted 6.1 M proteins from assemblies derived from 7.7 M Sanger sequences, and identified ∼39 k of these ‘20+ member’ PCs from surface ocean waters [19]. These POV data represent the first marine *viral-fraction* metagenomes analyzed using PC techniques (but see also [33], [47]), and they more than double the known viral PCs (Figure 2B). Specifically, the POV dataset ‘identified’ 12,302 GOS 20+ member PCs (these existed in GOS and a subset have been previously identified as viral [43], [48]), while adding 15,344 20+ member PCs represented only by POV sequences. These ∼28 k viral clusters are likely also abundant in nature as they represent only ∼6% of the total number of PCs (the ecological ‘binning’ unit), but recruit ∼68% of the POV ORFs (the ecological ‘count’ equivalent).

**Table 4:** Distribution and count by PC size.

PC size	# POV PCs	# POV reads	# POV ORFs	# GOS ORFs	# SIMAPphage ORFs
2–4	314 144	798 190	797 110	N/A	1 080
5–9	80 302	514 717	514 453	N/A	264
10–19	34 330	452 112	452 012	N/A	100
20–49	18 332	542 090	390 671	151 282	137
50–99	4 522	305 752	148 579	157 106	67
100–199	1 956	270 531	90 302	180 160	69
200–499	1 360	430 580	147 385	283 101	94
500–999	715	514 752	155 714	358 942	96
1000–1999	524	738 986	187 580	551 246	160
2000+	234	881 930	396 720	485 096	114
total	456 420	5 449 640	3 280 526	2 166 933	2 181

Distribution of PC sizes (based upon the number of ORFs a PC contains) and the number of PCs, POV reads mapping to clusters and ORFs that belonged to PCs of that size. All POV data are new to this study.

### Protein Clusters as a Viral Community Functional Richness Metric

Because viruses lack gene markers (e.g., small subunit ribosomal DNA) and most of the POV reads cannot be identified in reference databases measuring viral community diversity is problematic. To measure functional richness in POV samples irrespective of annotation, we detected genetic links between viral communities using protein clustering and illustrated patterns in richness between the samples using a rarefaction analysis as previously defined [33], [47].

#### Seasonal viral functional richness measurements at LineP

**Figure 3:**
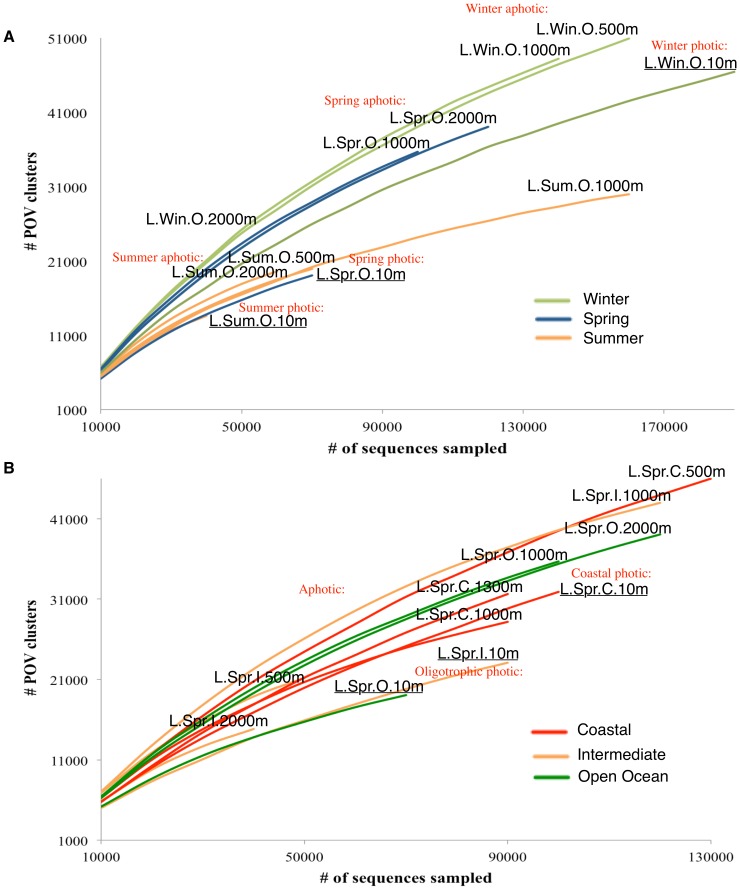
Viral community functional richness based on season and proximity to shore. Rarefaction analysis of hits to protein clusters from: (A) 11 POV metagenomes from a single LineP open ocean site (station P26) (B) 11 POV metagenomes from LineP stations P4, P12, and P26 from a single research cruise (June 2009). To be conservative, only protein clusters with >20 members were used in these analyses.

To examine ocean viral functional richness across the depth continuum (10 m to 2000 m) and season (spring, summer, and winter), 11 metagenomes from a single LineP open ocean site (station P26) were analyzed by protein cluster/rarefaction analysis [33], [47]. Rarefaction analysis showed that photic samples were less functionally rich than aphotic samples from the same season (Figure 3A). When comparing samples from different seasons in the same photic zone rarefaction analysis showed that winter was the most functionally rich, followed by spring, and summer (Figure 3A). All aphotic samples clearly separated by season, whereas photic samples showed similar levels of functional richness in spring and summer but increased richness in winter. Overall, rarefaction patterns indicate that season and photic zone are important drivers of viral community functional richness at LineP.

#### Viral functional richness measurements in June at LineP from coastal to open ocean

To examine viral functional richness across the depth continuum (10 m to 2000 m) and with proximity to shore (coastal vs open ocean), 11 metagenomes from the LineP transect stations P4, P12, and P26 from a single research cruise (June 2009) were analyzed by protein cluster/rarefaction analysis. Broadly, we found that aphotic samples from coastal to open ocean showed the same overall functional richness. Photic samples, however, were more functionally rich at the coast (station P4) as compared to oligotrophic intermediate and open ocean water samples (stations P12 and P26) that are similar in terms of their overall environmental chemistry. These data suggest that coastal photic waters are more functionally rich than photic open ocean waters, but the same pattern is not evident in the aphotic ocean.

**Figure 4:**
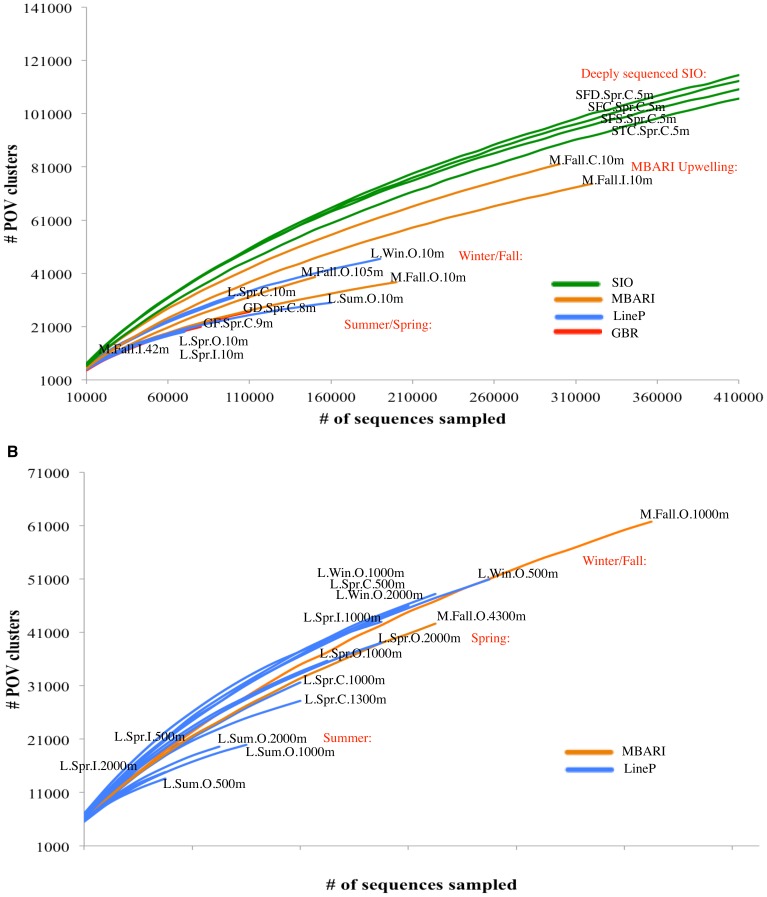
Viral community functional richness in the Pacific Ocean. Rarefaction analysis of hits to protein clusters from all POV metagenomes in (A) photic zone samples and (B) aphotic zone samples. To be conservative, only protein clusters with >20 members were used in these analyses.

Given that functional richness differed by photic zone in the smaller subset of LineP samples examined above, we analyzed the complete POV dataset by photic zone using a protein cluster/rarefaction analysis (Figure 4A and B). In the photic rarefaction analysis (Figure 4A), we found that deeply sequenced SIO samples were the most functionally rich. Though the rarefaction analysis should normalize the samples in terms of sequencing effort, the limitation we placed on our analysis to include only 20+ member clusters may have included rare SIO clusters that are highly represented due to the exceptional sequencing effort for this single sample. The samples with the next highest functional richness came from MBARI samples in the same current system as a local upwelling, followed by samples from fall/winter and spring/summer. We noted several exceptions to these general trends. First, the LineP spring coastal sample, L.Spr.C.10 m, grouped more closely to fall/winter samples, likely due to the higher functional richness noted previously in photic coastal samples. Secondly, the MBARI fall open ocean sample taken from waters in a deep chlorophyll maximum (DCM), M.Fall.O.105 m, grouped more closely to fall/winter samples, which could be due to increased functional richness in the DCM. Yet, we could not confirm this given the low sequencing effort and limited trend information in the rarefaction curve from other DCM sample, M.Fall.I.42 m. In the aphotic rarefaction analysis (Figure 4B), winter/fall/spring samples were the most functionally rich followed by summer.

In summary functional richness decreased (*i*) with distance from shore in surface waters only, (*ii*) from winter to summer, and (*iii*) from deep to surface waters. These data mirrored patterns seen in marine and brackish bacteria where samples decreased in diversity (*i*) along a river to ocean gradient [49], (*ii*) from winter to summer [50], (*iii*) and from deep to surface [49]. Our data provide a powerful new look at all of these features in a single analysis for viral metagenomes.

### Limitations of the POV Dataset

While taking great strides forward in providing a large-scale quantitative viral metagenomic dataset, POV is also not without biases and limitations. First, the dataset excludes ssDNA phages as these viral concentrates were purified using both DNase and cesium chloride banding (CsCl 1.35–1.5 g/ml), where ssDNA phages are in the lighter fractions [51], [52]. While most detectable viruses (DNA-stained counts suggested >99%) were in these fractions, it is now clear that ssDNA phages are often not detectable by such staining procedures [52]. Second, the dataset does not include RNA viruses as nucleic acid extractions were optimized for DNA and the linker amplification enzymes are specific for DNA. There are now methods which allow simultaneous purification of both RNA and DNA viruses [53], and already a road-map for constructing RNA viral metagenomes from the oceans [54]. However, protocols to isolate, purify, and amplify RNA for viral metagenomes have not been rigorously evaluated as was done for dsDNA viruses [31]–[34]. Third, the dataset excludes larger viruses as the source waters for viral community concentration were 0.22 µm pre-filtered to remove bacteria. Fourth, the average read length of ∼300 bp, while an improvement upon the other large-scale viral metagenomic datasets available, will undoubtedly be improved upon as sequencing technologies advance.

Finally, one sample is anomalously enriched for “bacteria” in the dataset. Specifically, while samples averaged 4.4%±3.7% bacterial hits per sample across the 32 sample dataset, a single sample showed elevated bacterial hits (24.1% for L.Spr.C.1000 m; Table 5; Figure S1). In the L.Spr.C.1000 m sample, 13.7% of reads matched bacteria from the family *Rhodobacteraceae* and 7.0% matched *Pseudoalteromonadaceae* representing the majority of bacterial hits (20.7% in these families as compared to 24.1% total). Further, the majority of reads (∼20% of total reads) from these two bacterial families mapped to just three genomes: *Sulfitobacter sp. EE-36*, *Pseudoalteromonas sp. SM9913, Sulfitobacter sp. NAS-14.1*, with 9, 7, and 4% of the hits, respectively. The locations of these “bacterial” reads in the reference genomes showed that the hits matched throughout the genomes indicating that these “bacterial” hits were not simply integrated phage DNA in the bacterial genomes, and must instead be either bacterial contamination that survived the DNase and CsCl purification steps or gene transfer agents (GTA, [55]–[57]). Given the extensive purification (cesium-chloride banding and DNase treatment) of the viral concentrate, we favor the hypothesis that this sample contains a higher GTA content than the rest of the samples. Regardless of the source of the microbial DNA, the L.Spr.C.1000 m sample should be used with caution, particularly for analyses related to auxiliary metabolic genes (AMGs, *sensu* [58]) or other genetic data known to co-occur in bacteria.

**Table 5:** Percentage of POV reads assigned to superkingdoms.

sample	viruses	bacteria	eukaryota	archaea	unknown
L.Win.O.1000 m	2.8%	2.9%	0.7%	0.0%	93.6%
L.Win.O.2000 m	2.5%	2.7%	1.0%	0.0%	93.8%
L.Spr.C.500 m	5.0%	4.0%	0.9%	0.0%	90.0%
L.Spr.C.1000 m	2.9%	24.1%	0.8%	0.0%	72.2%
L.Spr.C.1300 m	2.7%	2.5%	0.4%	0.0%	94.4%
L.Spr.I.500 m	5.2%	5.6%	1.3%	0.0%	87.9%
L.Spr.I.1000 m	4.0%	3.8%	0.9%	0.0%	91.3%
L.Spr.I.2000 m	5.9%	3.2%	0.7%	0.0%	90.2%
L.Sum.O.500 m	3.5%	4.3%	2.0%	0.0%	90.2%
L.Sum.O.1000 m	2.0%	2.9%	7.6%	0.0%	87.5%
L.Sum.O.2000 m	3.1%	4.1%	2.1%	0.0%	90.6%
L.Spr.O.1000 m	3.2%	2.9%	0.7%	0.0%	93.2%
L.Spr.O.2000 m	5.0%	3.7%	2.0%	0.0%	89.3%
L.Win.O.500 m	2.9%	2.7%	0.7%	0.0%	93.7%
M.Fall.O.1000 m	2.7%	3.4%	0.5%	0.0%	93.3%
M.Fall.O.4300 m	2.6%	2.9%	0.6%	0.0%	93.9%
GD.Spr.C.8 m	12.4%	4.6%	0.4%	0.0%	82.6%
GF.Spr.C.9 m	9.7%	4.5%	0.4%	0.0%	85.3%
L.Sum.O.10 m	4.5%	4.0%	0.9%	0.0%	90.6%
L.Spr.C.10 m	5.6%	2.7%	0.6%	0.0%	91.1%
L.Spr.I.10 m	4.7%	2.9%	1.2%	0.0%	91.2%
L.Spr.O.10 m	5.6%	5.1%	1.7%	0.0%	87.6%
L.Win.O.10 m	6.0%	3.3%	0.8%	0.0%	89.9%
M.Fall.C.10 m	8.0%	4.1%	0.7%	0.0%	87.2%
M.Fall.I.10 m	7.4%	3.4%	0.6%	0.0%	88.6%
M.Fall.I.42 m	5.3%	6.2%	2.8%	0.0%	85.8%
M.Fall.O.10 m	5.5%	3.5%	0.5%	0.0%	90.6%
M.Fall.O.105 m	15.3%	3.1%	0.4%	0.0%	81.2%
SFC.Spr.C.5 m	10.7%	4.7%	0.9%	0.0%	83.6%
SFD.Spr.C.5 m	9.1%	3.6%	0.7%	0.0%	86.5%
SFS.Spr.C.5 m	9.0%	3.8%	0.9%	0.0%	86.2%
STC.Spr.C.5 m	7.3%	4.1%	0.7%	0.0%	87.9%

Percentage of POV reads that were taxonomically assigned based on matches to proteins in SIMAP and curated as described in the methods by sample.

### Conclusions

Over the last two decades, viruses have emerged as abundant, diverse and biogeochemically important members of nearly any ecosystem. In spite of this importance, mapping the ocean virome has been stifled by technical challenges, limited sampling opportunities, and lack of database and analytical resources. The quantitative dataset and PC organization documented here provide an invaluable mapping resource for future comparative viral metagenomic research. Looking forward, marine viral ecology stands at a tipping point wherein the “unseen” majority [13] can now be spatiotemporally documented using large-scale metagenomic sequencing at a reduced cost. These ever-growing datasets, in combination with emerging information on novel phyla of microbial hosts [59], [60] and transformative experimental methods (e.g., single viral genomics [61], microfluidic digital PCR [62], viral tagging [63], phageFISH [64]) and *k-mer-based* annotation techniques to rapidly assign function [65] offer new windows into viral diversity across spatial and temporal scales that can be inter-connected with paired microbial datasets to link viruses and their hosts. Although the datasets and analyses are formidable, the curated PC dataset provided here should ease future adventures into the ‘unknown’ and lead to a better understanding of the dynamic microbial and viral processes that drive the biogeochemistry that fuels the planet.

## Materials and Methods

### Sample Collection, DNA Isolation, Linker Amplification, and Purification

Each sample, from ∼20–50 L of seawater, was pre-filtered using a 150 µm GF/A filter followed by a 0.22 µm, 142 mm diameter Express Plus filter. All filtrates were concentrated by FeCl_3_-precipitation and purified by DNase+CsCl. Comparison samples from SIO also had additional treatments as follows: (*i*) Tangential Flow Filtration (TFF) and DNase+CsCl, (*ii*) FeCl_3_-precipitation and DNase only, and (*iii*) FeCl_3_-precipitation and DNase+Sucrose as previously described [33]. DNA was extracted from the concentrated and purified viral particles using Wizard PCR DNA Purification Resin and Minicolumns as previously described [66]. The resulting DNA samples were randomly sheared and amplified using linker amplification (LA) as described previously [34].

Linker-amplified VLP DNA samples were sequenced using GS FLX Titanium sequencing chemistry on a 454 Genome Sequencer (http://www.454.com). Sequences were quality filtered using a custom pipeline written in Perl and bash shell and executed on a high performance computer running PBSPro to distribute jobs (screenpipe.tar). Briefly sequences were removed that (*i*) had an “N” anywhere in the sequence, and (*ii*) deviated from two standard deviations from the mean length or read quality score using protocols proposed by Huse *et al.* [67]. Artificial duplicates were removed from the pyrosequencing runs using the program cdhit-454 version 4.5.5 with default parameters [68]. All sequences were deposited to CAMERA (http://camera.calit2.net) under the following project accessions: CAM_P_0000914 and CAM_P_0000915.

### Assembly and ORF Finding

Metagenomic assembly and ORF calling was conducted using a custom pipeline written in Perl and bash shell and executed on a high performance computer running GridEngine to distribute jobs (ivelvet2_orfpipeline.tar). First, we removed singletons by finding reads that had a 20-mer frequency equal to zero using the vmatch package version 2.1.5 (kmer size = 20; http://www.vmatch.de/), because by definition they cannot contribute to overlap in an assembly [69]. Second, to skew the assembly towards assembling dominant species first and less dominant members in subsequent rounds of assembly, velvet version 1.0.15 (hash length = 29, -long) [70] was used to iteratively assemble sequences based on their k-mer frequency in 2+, 4+, 6+, 10+ bins. Third, the contigs from each frequency bin were merged to remove exact duplicates using cdhit version 4.5.5 and requiring a percent identity of 100% across the smallest contig [68]. Finally, the non-redundant maximally assembled contig dataset was used as input for ORF prediction using the metagenomic mode in Prodigal [71] along with the individual reads. All ORFs that were non-redundant and >60 amino acids in length were retained for further analysis similar to GOS [19].

### Protein Clustering

ORFs were clustered based on sequence similarity in a two-step process using cd-hit version 4.5.5 [68] using a custom pipeline written in Perl and bash shell and executed on a high performance computer using GridEngine to distribute jobs (protuniversepipeline.tar). First, ORFs were mapped to known PCs from the global ocean survey (GOS; [19]) and phage known protein sequences using cd-hit-2d (‘-g 1 -n 4 -d 0 -T 24 -M 45000’; 60% percent identity and 80% coverage). The proteins included in this initial clustering included GOS core cluster proteins (http://camera.calit2.net) and 33,857 proteins (NCBI) from known phage genomes downloaded on July 7, 2011 that were mapped to the associated SIMAP proteins for additional annotation information. Second, ORFs that did not match to known GOS clusters or phage genes from SIMAP were self-clustered using cd-hit as above. All ORFs, PCs and annotation are available as a public resource on the CAMERA website (http://camera.calit2.net) under the project accession: CAM_P_0000915.

### Taxonomic Classification

BLASTX [72] was used to assign taxonomy to ORFs and sequence reads by comparison to the Similarity Matrix of Proteins (SIMAP, [45], June 25^th^, 2011 release) using an analysis pipeline written in Perl and bash shell and executed on a high performance computer using PBSPro to distribute jobs (blastpipeline_simap.tar). Taxonomy was assigned based on the species taxonomy ID listed in SIMAP and at the superfamily, family and genus levels using the NCBI taxonomy hierarchy for that species ID. Data curation consisted of re-assigning hits to “uncultured” organisms to their next top match, as well as examining missing family and genus level data to create a curated a subset of the NCBI taxonomy records for the most abundant viruses [33].

### Rarefaction Analysis

All high quality metagenomic reads in the POV dataset (Table 2) were compared to ORFs in the 20+ member protein clusters using BLASTX (E value <0.001). We then generated hit counts to the protein clusters and used the data for further rarefaction analysis using the rarefaction calculator: (http://www.biology.ualberta.ca/jbrzusto/rarefact.php).

All protocols are available at http://eebweb.arizona.edu/Faculty/mbsulli/protocols.htm, and scripts and associated documentation are archived at http://code.google.com/p/tmpl/.

## Supporting Information

Figure S1 **Family taxonomic profile across POV samples by photic zone.** Note that these data represent only those metagenomic reads that had a significant hit to the SIMAP database.
(TIFF)

Figure S2 (A) Genus and (B) species taxonomic profile for all POV samples combined by photic zone. Note that these data represent only those metagenomic reads that had a significant hit to the SIMAP database.
(TIFF)

Table S1 **Marine Phage Genomes in NCBI Genbank as of November 2012.**
(DOCX)
