# Supplementary figures and images for: The Pacific Ocean Virome (POV): A Marine Viral Metagenomic Dataset and Associated Protein Clusters for Quantitative Viral Ecology
Source: PLoS One. 2013 Feb 28;8(2):e57355. doi: 10.1371/journal.pone.0057355 (PMC3585363; doi:10.1371/journal.pone.0057355)

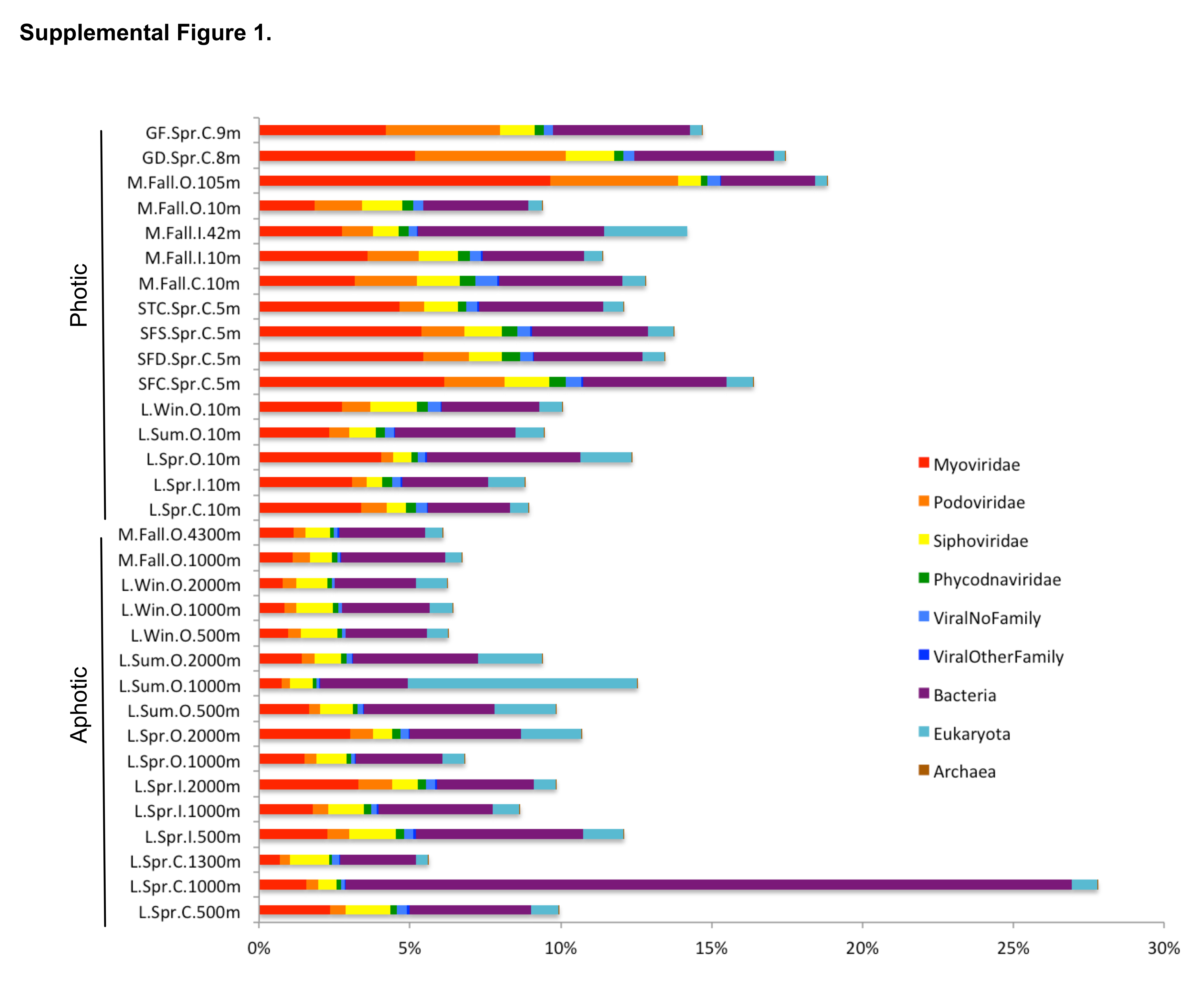

Supplement: Figure S1 — Family taxonomic profile across POV samples by photic zone. Note that these data represent only those metagenomic reads that had a significant hit to the SIMAP database. (TIFF) [file pone.0057355.s001.tiff]

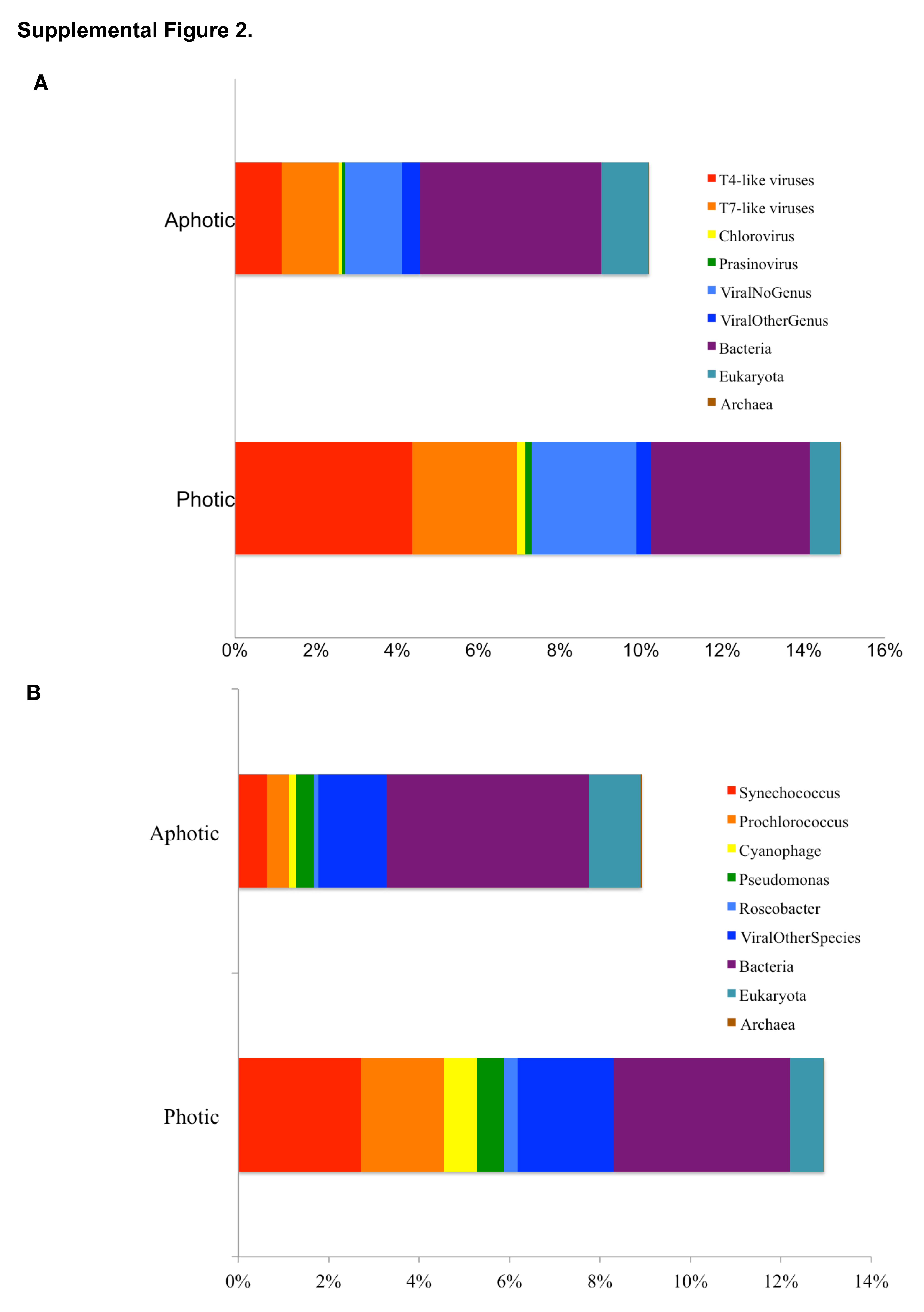

Supplement: Figure S2 — (A) Genus and (B) species taxonomic profile for all POV samples combined by photic zone. Note that these data represent only those metagenomic reads that had a significant hit to the SIMAP database. (TIFF) [file pone.0057355.s002.tiff]
