# Supplementary material for: The Pacific Ocean Virome (POV): A Marine Viral Metagenomic Dataset and Associated Protein Clusters for Quantitative Viral Ecology
Source: PLoS One. 2013 Feb 28;8(2):e57355. doi: 10.1371/journal.pone.0057355 (PMC3585363; doi:10.1371/journal.pone.0057355)
Supplement: Table S1 — Marine Phage Genomes in NCBI Genbank as of November 2012. (DOCX) [file pone.0057355.s003.docx]

Supplemental Table 1. Marine Phage Genomes as of November 2012.

| **Marine Phage Genome** | **Accession** | **Date** | **Group** | **Size (bp)** |
| --- | --- | --- | --- | --- |
| Aeromonas phage 25 | NC_008208 | 20060605 | myoviridae | 161,475 |
| Aeromonas phage 31 | NC_007022 | 20050406 | myoviridae | 172,963 |
| Aeromonas phage 65 | NC_015251 | 20101124 | myoviridae | 238,589 |
| Aeromonas phage Aes012 | JN377895 | 20120731 | myoviridae | 161,978 |
| Aeromonas phage Aes508 | JN377894 | 20120905 | myoviridae | 160,646 |
| Aeromonas phage CC2 | JX123262 | 20120913 | myoviridae | 231,743 |
| Aeromonas phage phiAS4 | NC_014635 | 20120224 | myoviridae | 163,875 |
| Aeromonas phage phiAS5 | NC_014636 | 20120426 | myoviridae | 225,268 |
| Aeromonas phage phiAS7 | JN651747 | 20120223 | podoviridae | 41,572 |
| Aeromonas phage phiO18P | NC_009542 | 20071214 | myoviridae | 33,985 |
| Aeromonas phage PX29 | GU396103 | 20101124 | myoviridae | 222,006 |
| Aeromonas phage vB_AsaM-56 | JQ177063 | 20120725 | myoviridae | 43,551 |
| Colwellia phage 9A | NC_018088 | 20120530 | siphoviridae | 104,936 |
| Cyanophage 9515-10a | NC_016657 | 20101031 | podoviridae | 47,055 |
| Cyanophage NATL1A-7 | NC_016658 | 20101031 | podoviridae | 47,741 |
| Cyanophage NATL2A-133 | NC_016659 | 20101031 | podoviridae | 47,536 |
| Cyanophage P-SSP2 | NC_016656 | 20101031 | podoviridae | 45,890 |
| Cyanophage PSS2 | NC_013021 | 20091031 | siphoviridae | 107,530 |
| Cyanophage S-TIM5 | JQ245707 | 20120216 | myoviridae | 161,440 |
| Deep-sea thermophilic phage D6E | GU568037 | 20101205 | unknown | 49,335 |
| Flavobacterium phage 11b | NC_006356 | 20100922 | siphoviridae | 36,012 |
| Halomonas phage phiHAP-1 | NC_010342 | 20090417 | myoviridae | 39,245 |
| Listonella phage phiHSIC | NC_006953 | 20050604 | siphoviridae | 37,966 |
| Phage phiJL001 | NC_006938 | 20070514 | siphoviridae | 63,649 |
| Prochlorococcus phage P-HM1 | NC_015280 | 20111213 | myoviridae | 181,044 |
| Prochlorococcus phage P-HM2 | NC_015284 | 20111213 | myoviridae | 183,806 |
| Prochlorococcus phage P-RSM4 | NC_015283 | 20111213 | myoviridae | 176,428 |
| Prochlorococcus phage P-SSM2 | NC_006883 | 20111213 | myoviridae | 252,401 |
| Prochlorococcus phage P-SSM4 | NC_006884 | 20111213 | myoviridae | 178,249 |
| Prochlorococcus phage P-SSM7 | NC_015290 | 20111213 | myoviridae | 182,180 |
| Prochlorococcus phage P-SSP7 | NC_006882 | 20120601 | podoviridae | 44,970 |
| Prochlorococcus phage Syn1 | NC_015288 | 20111213 | myoviridae | 191,195 |
| Prochlorococcus phage Syn33 | NC_015285 | 20111213 | myoviridae | 174,285 |
| Pseudoalteromonas phage PM2 | NC_000867 | 20100326 | corticoviridae | 10,079 |
| Psychrobacter phage Psymv2 | JF270478 | 20121007 | unknown | 35,725 |
| Rhizobium phage 16-3 | NC_011103 | 20100315 | siphoviridae | 60,195 |
| Roseobacter phage MB-2001 | FJ867914 | 20091029 | podoviridae | 38,252 |
| Roseobacter phage OS-2001 | FJ867913 | 20091029 | podoviridae | 38,205 |
| Roseobacter phage SBRSIO67-2001 | FJ867912 | 20091029 | podoviridae | 38,150 |
| Rhodobacter phage RcapMu | NC_016165 | 20111110 | siphoviridae | 39,283 |
| Rhodobacter phage RcapNL | JQ066768 | 20120219 | unknown | 40,489 |
| Roseobacter phage RDJL Phi 1 | NC_015466 | 20110426 | siphoviridae | 62,668 |
| Roseobacter phage SIO1 | NC_002519 | 20050415 | podoviridae | 39,898 |
| Silicibacter phage DSS3phi2 | NC_012697 | 20090810 | podoviridae | 74,611 |
| Sulfitobacter phage EE36phi1 | NC_012696 | 20090810 | podoviridae | 73,325 |
| Synechococcus phage P60 | NC_003390 | 20090417 | podoviridae | 47,872 |
| Synechococcus phage S-CBS1 | NC_016164 | 20120210 | siphoviridae | 30,332 |
| Synechococcus phage S-CBS2 | NC_015463 | 20120210 | siphoviridae | 72,332 |
| Synechococcus phage S-CBS3 | NC_015465 | 20120210 | siphoviridae | 33,004 |
| Synechococcus phage S-CBS4 | NC_016766 | 20120210 | siphoviridae | 69,420 |
| Synechococcus phage S-CRM01 | NC_015569 | 20110720 | myoviridae | 178,563 |
| Synechococcus phage S-MbCM6 | JN371768 | 20120313 | myoviridae | 176,043 |
| Synechococcus phage S-PM2 | NC_006820 | 20100708 | myoviridae | 196,280 |
| Synechococcus phage S-RIM8 A.HR1 | JF974288 | 20120322 | myoviridae | 171,211 |
| Synechococcus phage S-RIM8 A.HR5 | HQ317385 | 20120322 | myoviridae | 168,327 |
| Synechococcus phage S-RSM4 | NC_013085 | 20090922 | myoviridae | 194,454 |
| Synechococcus phage S-ShM2 | NC_015281 | 20111213 | myoviridae | 179,563 |
| Synechococcus phage S-SM1 | GU071094 | 20111213 | myoviridae | 174,079 |
| Synechococcus phage S-SM2 | GU071095 | 20111213 | myoviridae | 190,789 |
| Synechococcus phage S-SSM5 | GU071097 | 20111213 | myoviridae | 176,184 |
| Synechococcus phage S-SSM7 | GU071098 | 20111213 | myoviridae | 232,878 |
| Synechococcus phage S-ShM2 | GU071096 | 20111212 | myoviridae | 179,563 |
| Synechococcus phage Syn5 | NC_009531 | 20070508 | podoviridae | 46,214 |
| Synechococcus phage syn9 | NC_008296 | 20071209 | myoviridae | 177,300 |
| Synechococcus phage Syn19 | GU071106 | 20111213 | myoviridae | 175,230 |
| Thalassomonas phage BA3 | EU124666 | 20071007 | podoviridae | 37,313 |
| Vibrio phage 1 | JF713456 | 20120330 | siphoviridae | 81,509 |
| Vibrio phage CP-T1 | JQ177061 | 20120725 | myoviridae | 44,492 |
| Vibrio phage fs2 | AB002632 | 20090126 | unknown | 8,651 |
| Vibrio phage ICP1 | HQ641347 | 20110708 | myoviridae | 125,956 |
| Vibrio phage ICP1_2001_A | HQ641353 | 20110708 | myoviridae | 124,826 |
| Vibrio phage ICP1_2004_A | HQ641354 | 20110708 | myoviridae | 128,083 |
| Vibrio phage ICP1_2005_A | HQ641352 | 20110708 | myoviridae | 129,373 |
| Vibrio phage ICP1_2006_A | HQ641351 | 20110708 | myoviridae | 123,104 |
| Vibrio phage ICP1_2006_B | HQ641350 | 20110708 | myoviridae | 123,097 |
| Vibrio phage ICP1_2006_C | HQ641349 | 20110708 | myoviridae | 124,497 |
| Vibrio phage ICP1_2006_D | HQ641348 | 20110708 | myoviridae | 124,497 |
| Vibrio phage ICP2 | HQ641345 | 20110708 | podoviridae | 49,675 |
| Vibrio phage ICP2_2006_A | HQ641346 | 20110708 | podoviridae | 48,626 |
| Vibrio phage ICP3 | HQ641340 | 20110708 | podoviridae | 39,162 |
| Vibrio phage ICP3_2007_A | HQ641344 | 20110708 | podoviridae | 39,088 |
| Vibrio phage ICP3_2008_A | HQ641343 | 20110708 | podoviridae | 39,349 |
| Vibrio phage ICP3_2009_B | HQ641341 | 20110708 | podoviridae | 39,042 |
| Vibrio phage kappa | AB374228 | 20080116 | myoviridae | 33,507 |
| Vibrio phage K139 | NC_003313 | 20090417 | myoviridae | 33,106 |
| Vibrio phage KSF-1phi | NC_006294 | 20050607 | inoviridae | 7,107 |
| Vibrio phage KVP40 | NC_005083 | 20100326 | myoviridae | 244,834 |
| Vibriophage N4 | NC_013651 | 20120518 | podoviridae | 38,497 |
| Vibrio phage ND1-fs1 | AB572858 | 20120307 | inoviridae | 6,856 |
| Vibrio phage phiVC8 | JF712866 | 20110823 | podoviridae | 39,422 |
| Vibrio phage pVp-1 | JQ340389 | 20120603 | siphoviridae | 111,506 |
| Vibrio phage SIO-2 | HQ316604 | 20111120 | siphoviridae | 81,184 |
| Vibrio phage SSP002 | JQ692107 | 20120702 | inoviridae | 76,350 |
| Vibrio phage vB_VchM-138 | JQ177064 | 20120725 | myoviridae | 44,485 |
| Vibrio phage VCY-phi | JN848801 | 20111106 | inoviridae | 7,103 |
| Vibrio phage VEJphi | FJ904927 | 20100114 | inoviridae | 6,842 |
| Vibrio phage Vf12 | AB012574 | 20081213 | inoviridae | 7,965 |
| Vibrio phage Vf33 | AB012573 | 20081213 | inoviridae | 7,965 |
| Vibrio phage VfO3K6 | AB043678 | 20090127 | inoviridae | 8,784 |
| Vibrio phage VfO4K68 | AB043679 | 20090127 | inoviridae | 6,891 |
| Vibrio phage VHML | NC_004456 | 20100326 | myoviridae | 43,198 |
| Vibrio phage VP3 | JQ780163 | 20120411 | podoviridae | 39,481 |
| Vibrio phage VP5 | NC_005891 | 20050416 | podoviridae | 39,786 |
| Vibrio phage VP58.5 | FN297812 | 20100326 | myoviridae | 42,612 |
| Vibrio phage VP93 | FJ896200 | 20100421 | podoviridae | 43,931 |
| Vibrio phage VP882 | EF057797 | 20090501 | myoviridae | 38,197 |
| Vibriophage VpV262 | NC_003907 | 20030930 | podoviridae | 46,012 |
| Vibrio phage VSK | AF453500 | 20020212 | inoviridae | 6,882 |
| Vibrio phage VSKK | AF452449 | 20030812 | inoviridae | 6,834 |
| Vibrio phage VvAW1 | JQ801337 | 20120826 | unknown | 38,682 |
| Vibriophage phi-pp2 | JN849462 | 20120715 | myoviridae | 246,421 |
| Vibriophage VP4 | NC_007149 | 20050529 | podoviridae | 39,503 |
| Vibrio phage VP16C | AY328853 | unknown | myoviridae | 47,537 |
| Vibrio phage VP16T | AY328852 | unknown | myoviridae | 49,575 |
| Vibrio phage VP2 | NC_005879 | unknown | podoviridae | 39,853 |
